# Visual and Motor Deficits in Grown-up Mice with Congenital Zika Virus Infection

**DOI:** 10.1016/j.ebiom.2017.04.029

**Published:** 2017-04-24

**Authors:** Liyuan Cui, Peng Zou, Er Chen, Hao Yao, Hao Zheng, Qian Wang, Jing-Ning Zhu, Shibo Jiang, Lu Lu, Jiayi Zhang

**Affiliations:** a Institutes of Brain Science, State Key Laboratory of Medical Neurobiology, Collaborative Innovation Center of Brain Science, Key Laboratory of Medical Molecular Virology of Ministry of Education/Ministry of Health and Shanghai Public Health Clinical Center, Fudan University, Shanghai 200032, China; b Shanghai Mental Health Center, Shanghai Jiao Tong University School of Medicine, 600 Wan Ping Nan Road, Shanghai 200030, China; c State Key Laboratory of Pharmaceutical Biotechnology, Department of Biological Science and Technology, School of Life Sciences, Nanjing University, 163 Xianlin Avenue, Nanjing 210023, China

**Keywords:** ZIKV, Congenital Zika syndrome, Intracranial calcifications, Visual and motor dysfunctions, Prognosis evaluation

## Abstract

Human infants with congenital Zika virus (ZIKV) infection exhibit a range of symptoms including microcephaly, intracranial calcifications, macular atrophy and arthrogryposis. More importantly, prognosis data have lagged far behind the recent outbreak of ZIKV in 2015. In this work, we allow congenitally ZIKV-infected mice to grow into puberty. These mice exhibited motor incoordination and visual dysfunctions, which can be accounted by anatomical defects in the retina and cerebellar cortex. In contrary, anxiety level of the ZIKV-infected mice is normal. The spectrum of anatomical and behavioral deficits is consistent across different mice. Our data provided evidence that may help predict the public health burden in terms of prognosis of ZIKV-related congenital brain malformations in an animal model. Our study provided behavioral evaluation for the prognosis of congenital ZIKV infection and provides a platform for screening and evaluation of drugs candidates and treatment aiming at improving regeneration of infected neurons to prevent sequelae caused by ZIKV infection of fetus.

## Highlights

• Mice with congenital ZIKV infection manifest motor deficits in puberty may lead to paralysis.
• Mice with congenital ZIKV infection have unlaminated retina and distorted vision.
• This study has prognostic implication of human congenital ZIKV infection.

The recent outbreak of Zika virus and consequent increased number of babies with birth defects has raised great concern. However, knowledge about the prognosis of these children with congenital abnormalities has lagged behind. In this study, we raised congenitally ZIKV-infected mice into puberty. Our study revealed prominent motor and visual deficits as well as intracranial calcifications in the infected mice, which may help predict the public health burden.

## 1 Introduction

The first human case of Zika virus (ZIKV) infection was published in 1952 (Dick, 1952). However, up to 2007, only 14 human cases of ZIKV infection had been confirmed (Fagbami, 1979, Moore et al., 1975, Olson et al., 1981). Since late 2015, ZIKV infection has swept across > 35 countries and territories in Caribbean and South America (Fauci and Morens, 2016) after originating from an outbreak in Brazil (Campos et al., 2015). Although ZIKV has long been considered neurotropic (Dick, 1952, Dick et al., 1952), no specific neurological sequelae had ever been reported until this 2013–2014 epidemic during which reports linking ZIKV infection with Guillain-Barré syndrome emerged with noticeable frequency (Cao-Lormeau et al., 2016, Rasmussen et al., 2016). Of greater concern is an upsurge of cases and studies indicating a causal role between the ZIKV outbreak and an increased number of neonates in Brazil with birth defects, such as microcephaly and intrauterine growth restriction (IUGR) (Rasmussen et al., 2016, Heymann et al., 2016, Mlakar et al., 2016). However, up to now, we have gained very little knowledge about the prognosis of these children with congenital abnormalities due to the very recent outbreak of Zika.

Recent works simulated the vertical transmission of ZIKV in pregnancy and imitated its pathogenesis in fetuses (Cugola et al., 2016, Miner et al., 2016a, Wu et al., 2016, Li et al., 2016a). All the mice in these studies exhibited the microcephalic phenotype that was found in humans, and thus supported the causality between congenital ZIKV infection and microcephaly. However, none of the mice in these studies grew into puberty or adulthood either because of death of infected pups shortly after delivery (Li et al., 2016b) or the experimental necessity of ceasing the mice-rearing for further examinations. Therefore, the implications that could have otherwise been gained from these mouse models were, instead, limited to the mere link between microcephaly and ZIKV infection, not prognosis.

Although microcephaly was the most remarkable phenotype, it is not the only phenotype discovered in presumed ZIKV-infected human fetuses, as pointed out by many studies (França et al., 2016). In addition, evidence was advanced that the disease could be complicated by other clinical manifestations, such as IUGR (Brasil et al., 2016), intracranial calcification (ICC) (Oliveira Melo et al., 2016), cerebellar hypoplasia (de Fatima Vasco Aragao et al., 2016), arthrogryposis (van der Linden et al., 2016), and ocular malformations (Miranda et al., 2016), which, in the aggregate, constituted the so-called ‘congenital Zika syndrome’. Among these, the most distinctive is ICC, essentially because it was extensively believed to underlie the pathogenesis of congenital ZIKV infection (Livingston et al., 2014). Vision-threatening lesions including macular lesions and optic nerve abnormalities were recently reported to be clinically associated with congenital ZIKV-infection (de Paula Freitas et al., 2016). Arthrogryposis was present in the arms and legs of ZIKV-infected children (van der Linden et al., 2016). To date, these additional features show more and more conspicuous importance in the context of the ZIKV epidemic.

## 2 Materials and Methods

### 2.1 Zika Virus Preparation

Zika virus strain SZ01 (GenBank accession number: GEO: KU866423) used in this study was isolated from a Chinese male patient returning from Samoa to Shenzhen in February 2016 (Deng et al., 2016) and kindly provided by Dr. Cheng-Feng Qin. Virus stocks were amplified in mosquito C6/36 cells. The virus containing supernatants were clarified by centrifugation and stored at − 80 °C. The titer of ZIKV was determined by standard plaque-forming assay on BHK21 cells. Heat inactivation of ZIKV strain SZ01 was performed at 121 °C for 30 min.

### 2.2 Mice and Intra-amniotic Injection (IAI)

The pregnant C57BL/6 mice (E15) were purchased from Shanghai Yison Biotechnology Company.

Pregnant mice were anesthetized during the surgery at room temperature. Mouse belly was shaved and disinfected with alcohol and iodophor. A sterile gauze was draped over the abdomen and a slit was made to expose the abdominal incision. A 3 cm long laparotomy was carefully made around the position of womb. Firstly, abdominal skin was cut open along ventral midline, and peritoneum incision was performed carefully to avoid bleeding. The uterus was pulled out carefully using tweezers and kept wet with warm, 0.1 M sterile phosphate-buffered saline (PBS). 0.1 mL 250 and 500 PFU (Plaque-Forming Units) ZIKV SZ01 was inoculated into amniotic fluid of each embryo of 3 pregnant mice (in each group) through the uterine wall with pipette (World Precision Instrument, Inc., USA) pulled by micropipette puller (Sutter Instrument, USA), respectively. The pipette stayed in amniotic fluid for 10 s after injection to avoid virus overflow. 500 PFU of heat-inactivated ZIKV were injected in each embryo of another 10 pregnant mice, referred to as mock group. The uterus was repositioned into the womb after injection and the abdominal wall and skin were stitched. Lidocaine was applied on the wound. Mice were removed into cages to recover. All mice used in the experiments were from multiple litters. The survival rate for ZIKV-infected mice was 76% (29/38 pups), whereas that for mock-infected mice was 65% (24/37 pups). The difference in the survival rate between ZIKV-infected and mock group showed no significant difference (We assumed the distribution of survival rate is independent normally distributed. In Chi-square test, *n* = 75, expected values > 5, *P* = 0.2762). All mouse experimental procedures were approved by Institutional Animal Care and Use Committee at Shanghai Public Health Clinical Center. All measurements were conducted blind to the group.

### 2.3 RNA Extraction and Real-time Polymerase Chain Reaction (RT-PCR)

The total RNA was extracted from the brains or eyes of the pups with TRIzol Reagent (Invitrogen) according to the protocol provided by manufacturer. The RNA in mice urine was extracted by using QIAamp Viral RNA Mini Kit (Qiagen). The viral RNA copies were determined by RT-PCR (TaqMan) assay using One Step PrimeScript™ RT-PCR Kit (Takara). Primers and fluorogenic probes for ZIKV detection, ZIKV 835, ZIKV 911c and ZIKV 860-FAM were kindly provided by Prof. Yunwen Hu and Fahui Da. Reverse transcription reaction of 10 min at 42 °C was followed by PCR amplification using a 55 °C annealing temperature for 40 cycles in an ABI ViiA7 Real-Time PCR System. The standard curve of viral RNA copies was determined from 10-fold dilutions of plasmid containing ZIKV sequence (also kindly provided by Prof. Yunwen Hu and Fahui Dai) with known concentrations, and viral RNA copies were calculated.

### 2.4 Micro-computed Tomography (Micro-CT)

Mice were anesthetized and immobilized by adhesive tape. All micro-CT data were acquired using high-resolution, small animal imaging scanner (eXplore Locus, GE Healthcare, Port Washington, NY) with 80 kV, 450 μA and 100 ms exposure time. The detector bin mode was 4 × 4 and the effective pixel size was 0.092 mm over 360° of a total of 400 views, 3 frames per view. The raw data were converted into a VFF format compatible with MicroView ABA2.2 (GE Healthcare) to view intracranial calcification, measure the parameters of brain sizes and reconstruct 3D brains. We calculated the number of calcified foci for the whole brain and measured the lengths along both the major and perpendicular axis of the largest calcified foci of each brain in the sagittal sections. We measured the largest distances of dorsal-ventral (cranial height) and posterior–anterior (skull length) boundaries in sagittal sections, as well as medial-lateral (biparietal) in coronal sections. To calculate the brain volumes, we manually selected one out of every 5 sagittal sections in 2D Region of Interest (ROI) and reconstructed all the 2D images into 3D using the Advanced ROI Tool in MicroView ABA2.2.

### 2.5 Open Field Test

The open field (OF) test was used to assess the adaption and anxiety-like behavior as described previously (Blokland et al., 2002). Each mouse was placed in the center of a darkened white box (30 cm × 30 cm × 40 cm) and monitored by an infrared video tracking system for 25 min (Ethovision XT 9.0, Noldus Information Technology, The Netherlands). A 15 cm × 15 cm square in the center of the box was defined as Zone and the periphery arena as Residual. The distance traveled and time spent in the Zone and Residual were quantified for analysis.

### 2.6 Tail Suspension Test

Each mouse was suspended from a hanging hook located 45 cm above the bottom of the box using adhesive tape placed approximately 1 cm from the tip of the tail. Each mouse was tested for 6 min and recorded by a digital camera. Software (Ethovision XT 9.0, Noldus Information Technology, The Netherlands) was used to calculate the inactive (hanging passively without any struggle) time of each mouse.

### 2.7 Rota-rod Test

Each mouse was placed on a rotating spindle that accelerated from 4 revolutions per minute (rpm) to 40 rpm in 5 min (Rotamex-5, Columbus Instruments, Columbus, OH). All animals did one session per day for 3 days, and the latency to falling off the spindle in each session was recorded for analysis.

### 2.8 CatWalk

Each mouse walked freely on a glass lit by green light in the CatWalk system (CatWalk XT 10.0, Noldus Information Technology, The Netherlands). The gait and paw print images of each mouse were recorded by a high-speed camera for at least 3 sessions. Walking patterns and postures were analyzed.

### 2.9 Elevated Plus Maze

The apparatus consisted of two open arms (30 cm × 5 cm, 350 lx) and two close arms (30 cm × 5 cm × 15 cm, 20 lx), 50 cm from the ground. Each mouse was placed in the central platform (5 cm × 5 cm) facing an open arm and was allowed to move freely for 5 min Number of entries, distance traveled and time spent in open and close arms were analyzed (ANY-maze, Stoelting, Inc., Wood Dale, IL).

### 2.10 Immunohistochemistry

Mice were anesthetized and perfused with PBS followed by freshly made 4% paraformaldehyde (PFA, Sigma, P6148, US). Brain and retina were dissected. Brains were further fixed in 4% PFA overnight at 4 °C and then kept in 30% sucrose for dehydration. Retinas were further fixed in 4% PFA for 4–6 h at 4 °C and then kept in 10%, 20% and 30% sucrose successively for gradient dehydration. The tissue was embedded in optimal cutting temperature (OCT) compound (Leica, UK) after sedimentation in sucrose and stored at − 80 °C. Coronal or sagittal, 30 μm-thick brain slices and 14 μm-thick retina slices were sectioned with a freezing microtome (Leica SM 1950, Leica) and washed using Tris-buffered saline (TBS) 5 times (5 min each time) to remove the OCT. Sections were immersed in 0.5% Triton X-100 for 20 min followed by incubation for 2 h in 10% donkey serum in TBS (DST) incubation for 2 h at room temperature. Brain slices and retina slices were incubated with primary antibodies (FoxP2 (Santa Cruz Biotechnology, Cat# sc-21069, RRID: AB_2107124,1:200), FoxP1 (Abcam, Cat# ab16645, RRID: AB_732428, 1:2000), Ctip2 (Abcam, Cat# ab18465, RRID: AB_2064130, 1:1000), ChAT (Millipore, Cat# AB144P, RRID: AB_90650, 1:500), S-opsin (Santa Cruz Biotechnology, Cat# sc-14,365, RRID: AB_2236550, 1:800), Calbindin (Swant, Cat# CB-38a, RRID: AB_10000340, 1:1000), Brn3a (Santa Cruz Biotechnology, Cat# sc-31984, RRID: AB_2167511, 1:500)) for 20 h at 4 °C and then washed with TBS for 5 times (5 min each time). Sections were incubated with secondary antibodies (donkey anti-goat 488 (Jackson, Cat# 705-545-003, RRID:AB_2340428, 1:300), donkey anti-rat555 (Jackson, Cat# 712-165-153, RRID: AB_2340667, 1:200), donkey anti-rabbit 647 (Jackson, Cat# 711-605-152, RRID: AB_2492288, 1:200), and donkey anti-rabbit 488 (Jackson, Cat# 711-545-152, RRID: AB_2313584, 1:200) for 2 h at room temperature in the dark followed by Diamidino-phenyl-indole (DAPI) (Invitrogen, 1:3000) staining for 7 min, and then washed with TBS for 5 times (5 min each time). Brain slices were mounted onto slides and covered with coverslip. Slices were imaged using a Nikon microscope (Nikon Eclipse) or a confocal microscope (Nikon A1R).

### 2.11 Intravitreous Injection

Mice were anesthetized with 2% Chloral hydrate (0.02 mL per g weight). 1.0–1.2 μL cholera toxin subunit β (CTB) -555 (Molecular Probes, Invitrogen, C22843, US) were injected into left eye using NanoJectII (Drummond scientific company, Germany). To reduce overflow of the injected solution and equilibrate the intraocular pressure, the pipette was left in place for 10 s before withdrawal. After 36–48 h, mice were perfused and the brains were deal with same methods as immunohistochemistry.

### 2.12 Cell Counting

Threshold values of images with 8-bit greyscale format were adjusted to highlight the outlines of the cells in Image J. The images were made binary with only two intensities, black 0 and white 255. Watershed procedure served to separate overlapping cells. At last, cell number was calculated automatically while size parameter was set well from 300 to 5000 and circularity from 0 to 1 in Image J.

### 2.13 Data Analysis

Images were analyzed using Illustrator from Adobe. Statistical analysis was performed using Origin (OriginLab Corporation, US) and Matlab (MathWorks, US). All data were tested for normality using Shapiro-Wilk test before statistical analysis. The normally distributed data were analyzed with one-way ANOVA followed by *post hoc* Bonferroni test, Tukey test were performed to compare pairwise data. The data that did not distribute normally was analyzed with Kruskal-Wallis ANOVA. In all cases, the criterion for statistical significance was *p* < 0.05 and error bars represented SEM.

## 3 Results

### 3.1 Intra-amniotic Injection of ZIKV Caused Decrease in the Brain Volume and Cortical Thickness

Observations from earlier studies of congenital ZIKV infection show that embryos that were intra-ventricle infected at E13.5 do not survive after birth (Li et al., 2016b). We injected ZIKV and heat-inactivated (Mock) ZIKV into the amniotic fluid of embryos (E15) in C57BL/6 pregnant mice. A total of 86.4% (38/44 pups) of the injected pups were born, and 76.3% (29/38 pups) survived into adulthood, indicating that the intra-amniotic injection (IAI) had less impact on the embryos compared to intra-ventricular injection that is lethal for all the embryos (Li et al., 2016a). Among all titers, 500 PFU was most efficient at inducing anatomical and functional phenotypes.

**Fig. 1:**
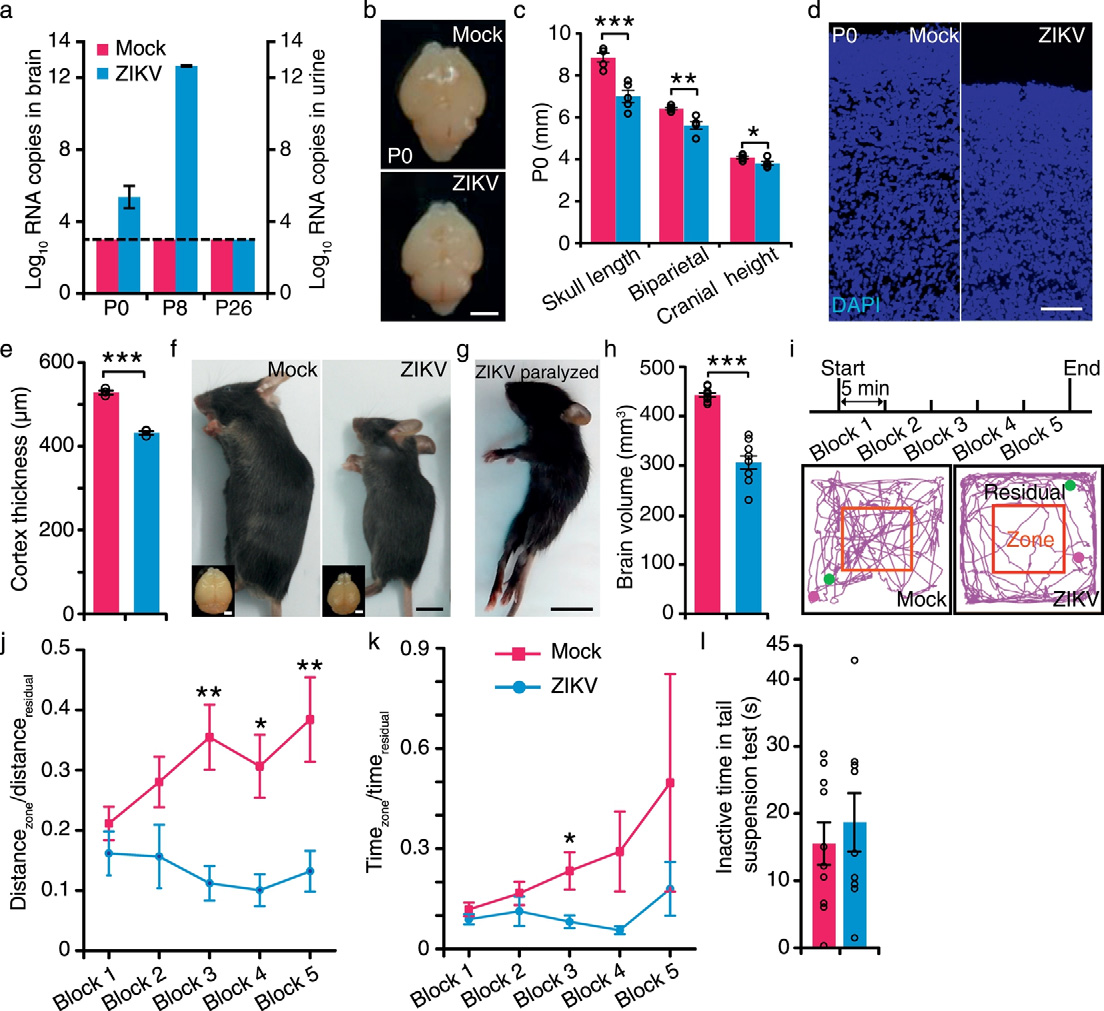
Intra-amniotic infection of Zika Virus in C57 mice and their behaviors in open field and tail suspension tests. (a) Viral RNA copies determined by real-time PCR of whole brains (P0, P8) and urine (P26) in mock- and ZIKV-infected mice. Dotted lines represent limits of detection (n_mock_ = 4 mice, n_ZIKV_ = 4 mice). (b) Whole mount brains of mock- and ZIKV-infected mice at P0. Scale bar, 2 mm. (c) Skull length, biparietal and cranial height of mock- and ZIKV-infected mice (n_mock_ = 5 mice, n_ZIKV_ = 5 mice). (d) DAPI (blue)-stained cortices of mock- and ZIKV-infected mice at P0. Scale bar, 100 μm. (e) Thickness of the cerebral cortices of mock- and ZIKV-infected mice at P0 (n_mock_ = 3 mice, n_ZIKV_ = 3 mice). (f) Mock- and ZIKV-infected mice. Scale bar, 1 cm. Insets in (f), their whole mount brains. Scale bar, 2 mm. (g) ZIKA-infected paralyzed mouse at P20. Scale bar, 1 cm. (h) Brain volume of mock-infected and ZIKV-infected mice measured from Fig. S1b. (i) Top: schematics for the open-field experiment. Bottom: traces of mock- (left) and non-paralyzed ZIKV-infected (right) mice in block 3 of open-field experiment. Red squares indicate the boundary between the zone and residual areas. Magenta dot and green dot indicated the start and end point respectively. (j and k) The ratio of distance traveled and time spent between zone and residual (n_mock_ = 10 mice, n_ZIKV_ = 9 mice). (l) The inactive time of mock- and ZIKV-infected mice in tail suspension test (n_mock_ = 10 mice, n_ZIKV_ = 9 mice). All the data showed mean ± SEM. *P < 0.05, **P < 0.01, ***P < 0.001. Black circles represented raw data.

Previous reports demonstrate a direct link between ZIKV infection and microcephaly in embryonic or neonatal mice and monkey (Adams Waldorf et al., 2016, Cugola et al., 2016, Miner et al., 2016a, Wu et al., 2016, Li et al., 2016a). We confirmed the ZIKV infection by detecting viral RNA copies in brains and urine of ZIKV-infected mice (Fig. 1a). The ZIKV-specific RNA could be detected in brains of ZIKV-infected mice at P0 and at P8, while no viral RNA was detected in the brain of mock-infected mice. The viral RNA copy number increased greatly by about ten million-fold from P0 to P8, which indicated the persistent infection and replication of ZIKV in the mice. When the mice grew up to puberty for behavioral tests, the viral RNA was undetectable in the urine at P26, suggesting that with the maturation of the immune system when the mice grew up, the virus could be eliminated. However, the persistent ZIKV infection in the infants has caused irreversible damage. We showed that brains of ZIKV-infected mice were smaller than those of mock-infected mice at P0 (the day of mice birth was defined as 0 postnatal day) (Fig. 1b and c) (Cugola et al., 2016, Miner et al., 2016a), suggesting growth restriction of ZIKV-infected mice at infant. Furthermore, ZIKV infection led to a thinner cerebral cortex at P0 (Fig. 1d and e). For those mice surviving ZIKV infection, body weight was reduced (Fig. S1a) and brain volume was significantly smaller compared to mock-infected mice at P40 (Fig. 1f and h). Micro-CT showed severely reduced brain size, skull length, biparietal diameter, and cranial height in ZIKV-infected mice (Fig. S1b–d). We used FoxP1 and FoxP2 (Li et al., 2016a) to label layer II-VI and V-VI cortical neurons, respectively. Cortices of motor, somatosensory and visual areas manifested reduction of thickness in almost all labeled layers (Fig. S2), which is reminiscent of the sign of malformation of cortical development (MCD) in microcephalic human cases (de Fatima Vasco Aragao et al., 2016). Consistent with previous reports (Li et al., 2016a), the lamination of cerebral cortex appeared to be normal (Fig. S2a–f‴). Subcortical structures such as striatum were also mostly normal (Fig. S3a–c). We further examined the hippocampus of ZIKV-infected mice. We found that the dentate gyrus of hippocampus exhibited hypoplasia (Fig. S3d–g).

### 3.2 Paralysis and Deficits in Motor Functions in ZIKV-infection Mice

Two mice exhibiting hind limb paralysis (Fig. 1g and Video S1), most likely from arthrogryposis (joint contractures), were excluded from the behavioral tests due to the immobility. To explore the behaviors of adult ZIKV-infected mice under natural circumstances, we put the mice in an open-field test in complete darkness (Fig. 1i). The results showed that the distance traveled and time spent in the central zone were similar in both ZIKV- and mock-infected mice in block 1 (Fig. 1j and k), suggesting a normal anxiety level in ZIKV-infected mice. However, they failed to travel more distance in the central zone from block 3 to block 5 (Fig. 1j and k), indicating desensitization to novelty. The tail suspension test showed that ZIKV-infected mice spent similar time in inactive state to control mice (Fig. 1l), consistent with the results in the open-field test that stress or anxiety is normal in ZIKV-infected mice.

**Fig. 2:**
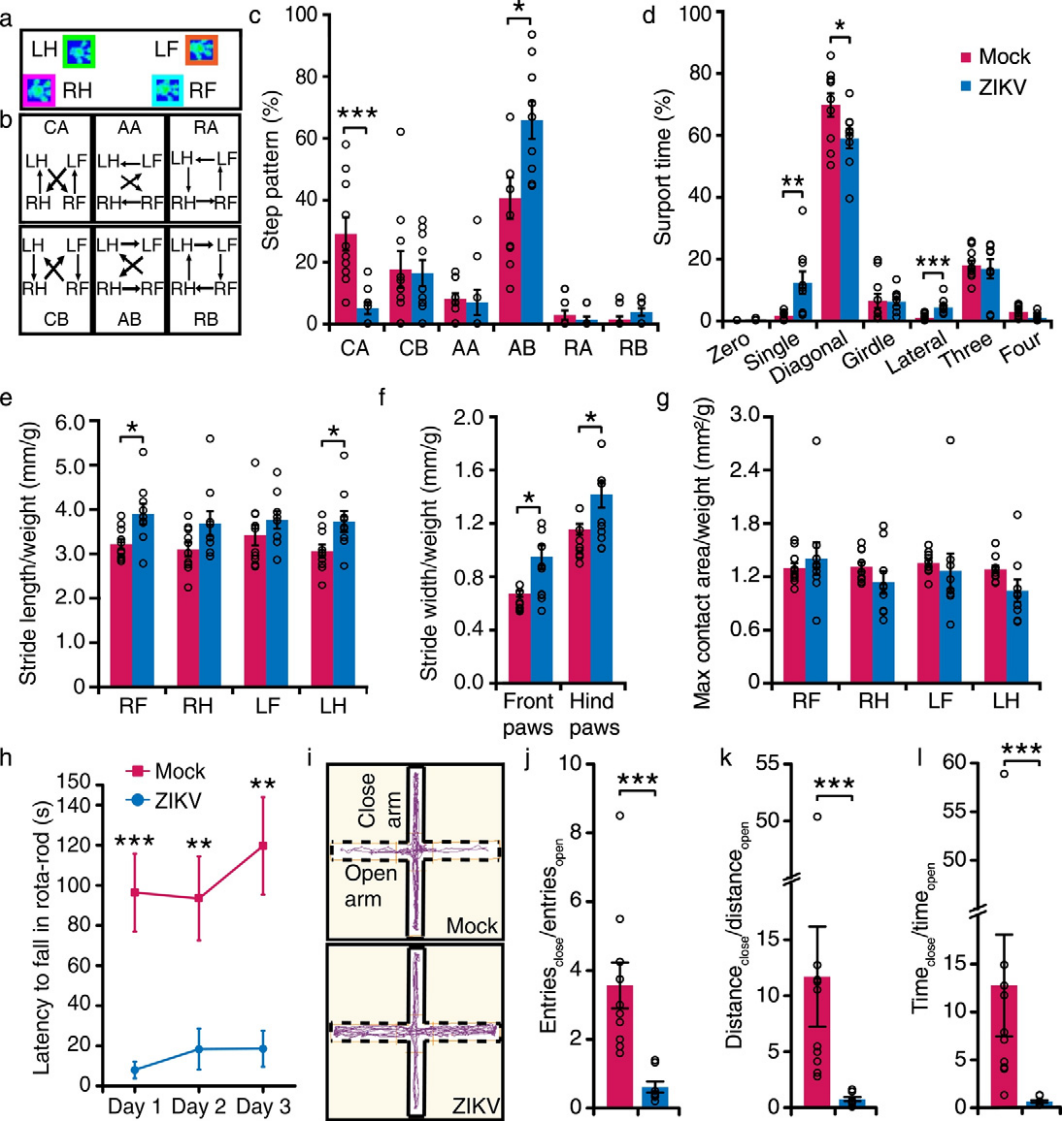
Motor and visual dysfunctions in ZIKV-infected mice. (a) Representative paw print images acquired by Catwalk system. LF: Left front; RF: Right front; LH: Left hind; RH: Right hind. (b) Schematic diagrams of six step patterns. (c and d) Percentage of different step patterns and support time spent from mock- and non-paralyzed ZIKA-infected mice. Zero: support without paws; Single: support with one paw; Diagonal: support with two diagonal paws; Girdle: support with both front paws or hind paws simultaneously; Lateral: support with two paws at the same side of the body; Three: support with three paws; Four: support with four paws (n_mock_ = 10 mice, n_ZIKV_ = 9 mice). (e–g) The ratio of stride length (e), stride width (f) and max contact areas (g) from print to body weight of mice (n_mock_ = 10 mice, n_ZIKV_ = 9 mice). (h) Latency to fall off the rota-rod of mock- and non-paralyzed ZIKA-infected mice at P40. (i) The traces of mock- and ZIKV-infected mice in elevated plus maze. Solid line: close arm; dotted line: open arm. (j–l) The ratio of number of entries (j), distance traveled (k), and time spent (l) in close and open arms (n_mock_ = 10 mice, n_ZIKV_ = 8 mice). All the data showed mean ± SEM, ***P < 0.001, **P < 0.01, *P < 0.05. Black circles represented raw data.

It is important to note that our pubertal ZIKV-infected mice model showed a remarkable deficit in motor functions. Many of the ZIKV-infected litter started to show walking defects by P20 (Video S2). We performed catwalk experiments at P40 to examine the gait and walking postures (Fig. 2a and b). ZIKV-infected mice had a smaller fraction of CA step pattern and larger fraction of AB step pattern than the mock-infected mice (Fig. 2c). In addition, ZIKV-infected mice spent much more time on single paw and lateral paws and less time on diagonal paws than control mice (Fig. 2d). The stride length and width were slightly different between ZIKV-infected and mock-infected mice (Fig. 2e and f), but the calibrated maximum contact area of the paws in ZIKV-infected mice was similar to that in mock-infected mice (Fig. 2g). This suggests the way of paws toughing ground in ZIKV-infected mice was normal. We conducted rota-rod test to further examine motor coordination and motor balance of ZIKV-infected non-paralyzed mice. The mice spent significantly shorter time on the rota-rod than control mice on day 1 through day 3 (Fig. 2h). Furthermore, unlike control mice, performance of ZIKV-infected mice on the rota-rod hardly improved during the 3-day training. In summary, motor functions of our ZIKV-infected mice were largely impaired, which is in line with motor deficit in congenital Zika syndrome.

To confirm the results from the tail suspension test, we also put the mice in an elevated plus maze in white light, which consisted of two elevated arms with closed walls and another two elevated open arms (Fig. 2i). Surprisingly, ZIKV-infected mice had a similar number of entries to both arms and spent a similar amount of time in both arms, whereas the control mice had a clear preference to linger in the arm with closed walls (Fig. 2j–l). Moreover, one out of eight ZIKV-infected mice fell from the open arm several times during the experiment. Another mouse even fell from the open arm at the very beginning of the experiment, and thus was excluded from the analysis. Given that congenital ZIKV infection causes macular lesions in children (de Paula Freitas et al., 2016), we speculate that the impaired vision of ZIKV-infected mice may account for their inability of differentiating the open arm from the close arm.

### 3.3 ZIKV-infection Caused Deficits in the Visual Circuits and Cerebellum as well as Intracranial Calcification

**Fig. 3:**
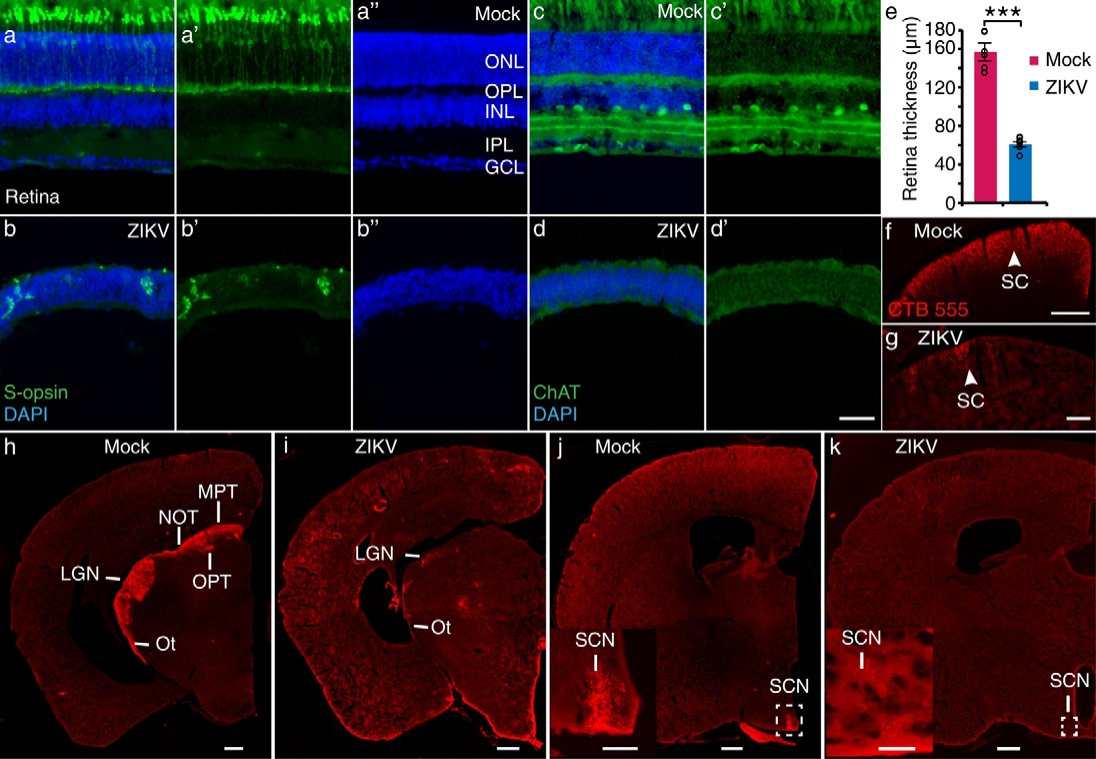
Defects in the visual circuits of ZIKV-infected mice. (a–b″) S-opsin (green) and DAPI (blue) stained retinas of mock- and ZIKV-infected mice. (c–d′) ChAT (green) and DAPI (blue) stained retinas of mock- and ZIKV-infected mice. Scale bar, 50 μm. (e) Retinal thicknesses of mock- and ZIKV-infected mice. n_mock_ = 6 mice, n_ZIKV_ = 6 mice, ***P < 0.001. CTB-555 (red) labeled retinal projection to the LGN (h and i) and SC (f and g) in mock- and ZIKV-infected mice. (h and i) CTB-555 (red) labeled retinal projections to OPT, Ot, MPT and NOT in mock- and ZIKV-infected mice. (j and k) CTB-555 (red) labeled retinal projections to SCN in mock- and ZIKV-infected mice. Scale bars, (f, g) 200 μm, (h–k) 500 μm. Insets in (j) and (k): high magnification images of white-dotted inset area in (j) and (k). Scale bars, inset in (j) 200 μm, inset in (k) 100 μm. ONL: outer nuclear layer; OPL: outer plexiform layer; INL: inner nuclear layer; IPL: inner plexiform layer; GCL: ganglion cell layer; LGN: Lateral geniculate nucleus; SC: Superior colliculus; OPT: Olivary pretectal nucleus; Ot: Optic tract; MPT: Medial pretectal nucleus; NOT: Nucleus of the optic tract; SCN: Suprachiasmatic nucleus. All data showed mean ± SEM. Black circles represented raw data.

Inspired by the results from elevated cross maze tests, we examined the histology of the visual system in ZIKV-infected mice. We found that retinas of ZIKV-infected mice exhibited no lamination in outer nuclear, inner nuclear and ganglion cell layers (Fig. 3a″ and b″), accompanied by reduced thickness at P40 (Fig. 3e). The expression of S-opsin, a short wave (blue) sensitive marker in retinal cone photoreceptors was dramatically decreased in ZIKV-infected mice than mock-infected mice (Fig. 3a-b′). We used choline acetyl transferase (ChAT) to label amacrine cells and their dendrites distributed in the ON and OFF layers of the retina, and no amacrine cell can be detected in ZIKV-infected mice at P40 (Fig. 3c-d′). The retinal lamination of ZIKV-infected mice at P12, however, was normal, despite a decrease in the thickness and number of photoreceptors, amacrine cells and retinal ganglion cells (labeled by Brn3a) (Fig. S4a–f″). These results suggest that the histological deficit in the retina were progressive. Meanwhile, the optic nerve was diminished severely at P12 (Fig. S4g–j). We also confirmed the ZIKV infection by detecting viral RNA copies in eyes of ZIKV-infected mice at P8 (Fig. S4k). The ZIKV-specific RNA could be detected in eyes of ZIKV-infected mice, while no viral RNA was detected in the eyes of mock-infected mice. We further injected cholera toxin subunit β (CTB)-dyes into one of the eyes to trace the retinal axons into the visual nuclei. Consistent with the lack of retinal ganglion cell layer in the retina, there was a severe reduction in retinal projections to both the lateral geniculate nucleus (LGN) and superior colliculus (SC) (Fig. 3f–i), suggesting impaired image-forming vision. Moreover, retinal projections to other visual related nuclei such as medial pretectal nucleus (MPT) and suprachiasmatic nucleus (SCN) were also abolished (Fig. 3h–k).

**Fig. 4:**
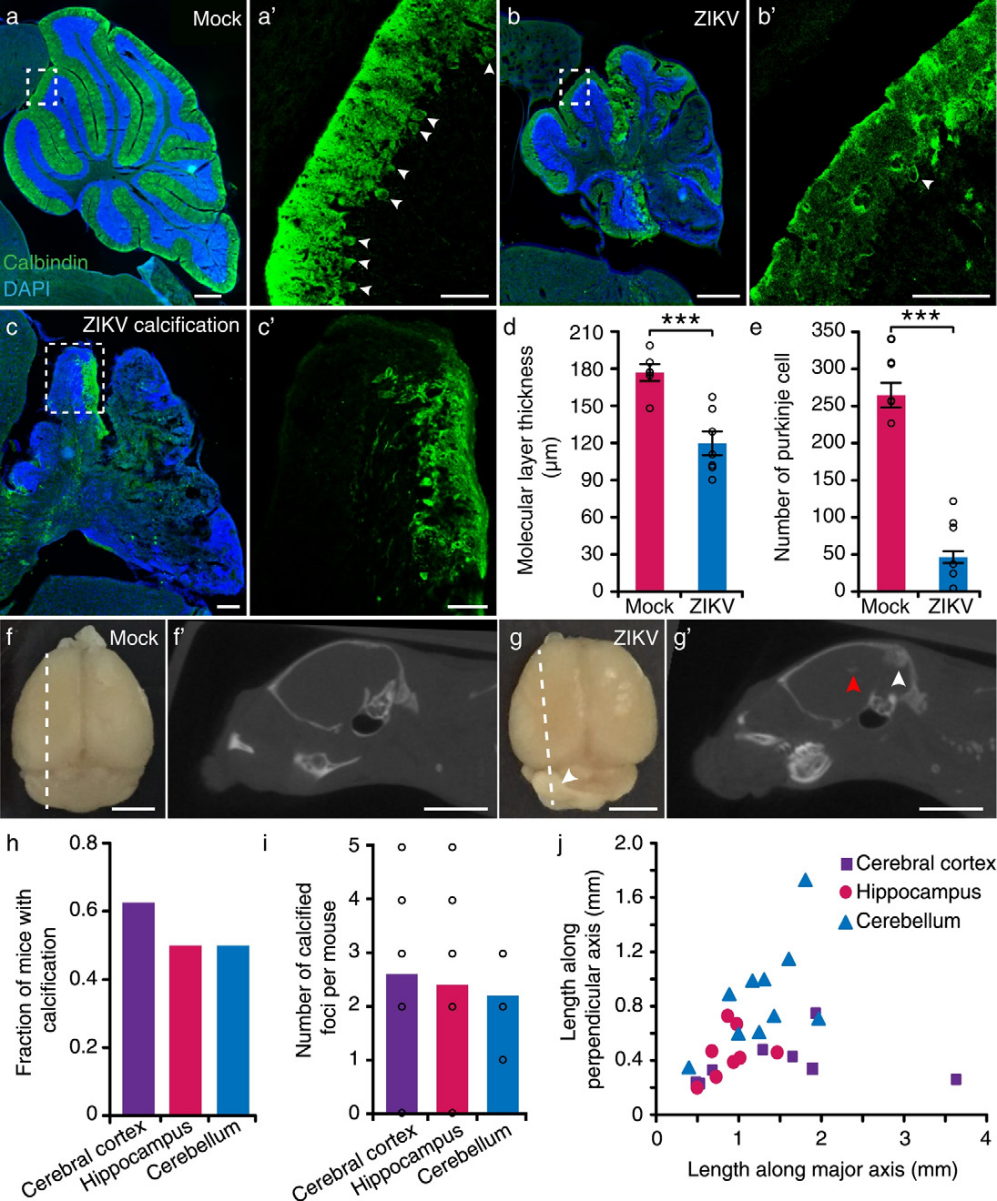
Cerebellar defects and intracranial calcification in ZIKV-infected mice. (a and b) Images of mock- and ZIKV-infected mice stained with Calbindin (green) and DAPI (blue). Scale bars, 500 μm. (c) Calbindin (green) and DAPI (blue) stained brain of a ZIKV-infected mice with severe calcification. Scale bar, 200 μm. (a′, b′ and c′) High magnification image of the dotted white box outlined in (a, b and c), respectively. Scale bars, 100 μm. (d and e) Thickness of molecular layers and the number of Purkinje cell in cerebellum (n_mock_ = 6 mice, n_ZIKV_ = 7 mice), ***P < 0.001. Images of mock- (f) and ZIKV-infected (g) brains at P40. Scale bars, 3 mm. (f′ and g′) Sagittal micro-CT images of mock- and ZIKV-infected mice, whose positions were indicated in white dotted lines in (f and g). Scale bars, 5 mm. White arrows: positions of calcified foci shown in (g). Red arrows: positions of calcified foci that did not appear in (g). (h and i) Fraction of mice that had calcification (*n* = 16 mice) and the average number of calcified foci per mouse (*n* = 10 mice) in the cerebral cortex, hippocampus and cerebellum, respectively. (j) Scatter plot of lengths along both the major and perpendicular axes of the largest calcified foci in each brain (*n* = 10 mice). All data showed mean ± SEM. Black circles represented raw data.

50% of patients with presumed congenital ZIKV infection had hypoplasia of cerebellum or brainstem (Soares de Oliveira-Szejnfeld et al., 2016). However, previous reports in ZIKV-infected embryonic mice did not emphasize on abnormalities in the cerebellum (Cugola et al., 2016, Li et al., 2016a). The sizes of the cerebellum in our ZIKV-infected mice were greatly reduced, accompanied by a reduction in the number of Purkinje cells as well as thickness of the molecular layer (Fig. 4a–b′, d and e). A total of 22% (2/9 mice) of ZIKV-infected mice had severe defects in cerebellum lamination, especially molecular layer and Purkinje cell layer in which no Purkinje cell body was identified (Fig. 4c and c′). Therefore, our results suggest a link between ZIKV infection and hypoplasia of the cerebellum, which may result in gait disturbance and motor incoordination observed in the ZIKV-infected mice.

Most patients with presumed congenital ZIKV infection had intracranial calcification (ICC) located mostly in the junction between cortical and subcortical white matter (de Fatima Vasco Aragao et al., 2016), which was not previously reported in neonatal ZIKV mouse models (Cugola et al., 2016). We next used micro-CT to study ICC in our ZIKV-infected mice. Calcification appeared as high-density areas on CT in various locations of the brain, including cortex and cerebellum. Fig. 4f–g′ showed one mice that had multiple calcified foci located in the cerebral cortex, hippocampus and cerebellum. A total of 62.5% (10/16) of the mice had calcifications in the cerebral cortex, with 2.6 calcified foci per mouse (Fig. 4h and i). The fraction of mice with calcifications in the hippocampus and cerebellum was smaller than that in the cerebral cortex (Fig. 4h). Moreover, each calcified foci was up to a few mm in size (Fig. 4j). No calcification was observed in mock-infected mice.

## 4 Discussion

Despite primates being the major host of ZIKV (Haddow et al., 1964, Hamel et al., 2016), early experiments suggested that mice could be infected by ZIKV through transmission of *Aedes aegypti* (Cornet et al., 1979, Darwish et al., 1983). Neurovirulence of ZIKV was also confirmed in mice (Dick, 1952). Thus, murine models of congenital ZIKV infection were the earliest in history to be successfully established (Mysorekar and Diamond, 2016). Various methods to induce ZIKV infection including intravenous injection, intraperitoneal injection, intraperitoneal injection and brain injection have thus far been reported (Cugola et al., 2016, Li et al., 2016a, Miner et al., 2016a, Wu et al., 2016). We established an immuno-competent murine model of congenital ZIKV infection using intra-amniotic injection. Most of these ZIKV-infective mice can survive to adulthood. Our study helped evaluate the prognosis of congenital ZIKV infection and provides a platform for screening and evaluation of drug candidates and treatment aiming at improving regeneration of infected neurons to prevent sequelae caused by ZIKV infection of fetus.

Recently, more and more researchers have begun to turn their attention to the extra-microcephalic signs and symptoms observed in ZIKV-infected children (Miranda et al., 2016, Melo et al., 2016). Many clinical studies examined ophthalmologic involvement, including chorioretinal atrophy and optic nerve abnormalities, in infants with presumed congenital ZIKV infection (de Paula Freitas et al., 2016, Jampol and Goldstein, 2016, Mccarthy, 2016). In addition, redundant scalp skin, arthrogryposis and clubfoot were also described previously (Rasmussen et al., 2016), which led to the coinage of the new term ‘congenital Zika syndrome’ (França et al., 2016, Melo et al., 2016). However, these additional but equally important manifestations have never been verified or reported in studies with animal models, although one study did mention panuveitis with no histological abnormalities of the eyes in their model (Miner et al., 2016b).

In our study, we described the severe histological deficits in the mouse retina with congenital ZIKV infection. We found that the retinas of ZIKV-infected mice were thinner with disrupted molecular lamination. Such changes, as those observed in our mouse model, are suggestive of the pathology that might underlie ocular findings in human cases, such as macular atrophy (de Paula Freitas et al., 2016). Retinal projections to the visual nuclei were also largely abolished in our ZIKV-infected mice, which probably implied that not only the normal migration of neural progenitor cells but also their subsequent axonal development was impaired (Lee et al., 2010). Furthermore, the frequent falls of ZIKV-infected mice from the open arms in the following elevated plus maze (EPM) experiment were also indicative of their visual impairments. Hence, our study provides an appropriate tool for further ophthalmological studies (Miranda et al., 2016). Retinal and cortical cells are considered to be homogeneous whose spontaneous activities are coordinated (Livingston et al., 2014), and many studies suggest that neural progenitor cells may be the major targets of ZIKV (Dang et al., 2016). Combined with our observation that retinal development was hampered, it is possible that the insult of ZIKV occurs before neural progenitor cells differentiate into the subgroups of retinal progenitor cells and encephalic cells.

Interestingly, we found that two congenitally infected ZIKV mice in our study exhibited a phenotype similar to arthrogryposis (joint contractures), as has been described in several clinical case reports (van der Linden et al., 2016). One recently published retrospective study summarized the clinical and radiological features of arthrogryposis in presumed ZIKV-infected children including the presence of arthrogryposis in all the four limbs of 86% of children, but only the lower limbs of 14% (van der Linden et al., 2016). It is possible that previous animal model studies found no such similar phenomenon because arthrogryposis is not remarkable at birth and, in contrast to our study, these studies did not rear neonatal mice to the age when mice learn to walk.

In our study, we examined motor functions of infected mice after reaching puberty. Their motor functions were severely influenced, as shown by gait analysis and the rota-rod experiment. These findings were reminiscent of our results in which we found that mouse cerebellum and motor cortex anatomy was abnormal. These results lead us to suspect that the motor defect of ZIKV-infected mice was at least partly, if not totally, a result of abnormal cerebella and motor cortices, considering the strong correlation between motor disorders and cerebellar defects (Malone et al., 2014). In Brazil, it is estimated that > 1800 babies were born with microcephaly. As these children grow up, they will, with no doubt, place a great burden on the health care system. However, the precise endpoint of congenital ZIKV infection remains unknown. Hence, the long-term prognosis of these children becomes a critical question in urgent demand of clinical surveys and experimental predictions.

In the past few months, our understanding of ZIKV and other flaviviruses has deepened to an unprecedented level. We provided the prognostic evidence about the motor and visual function impairments in mice with congenital ZIKV infection, which might help researchers and healthcare workers to predict the public health burden of the ‘Zika generation’ in countries hit by this rampant virus and devise further epidemiologic studies to evaluate the sequelae of this ZIKV epidemic. Meanwhile, our model, due to higher survival rate and more pups grown into puberty or adulthood, makes it possible to develop a prognostic evaluation system for behavioral analysis, which can be used to identify the antiviral effect of a drug candidate, as well as study drugs for repair and regeneration of the nervous system by using behavioral analysis. Especially, drugs and therapies, such as nerve growth factors, which do not inhibit virus infection, but rather benefit nerve repair, can be evaluated to determine if they improve the prognosis of an infant when administered either alone or in combination with anti-ZIKV drugs. These drugs and therapies are particularly useful for the pregnant women if ZIKV infection is detected in the later stage of gestation, making abortion unacceptable.

In summary, we develop an intra-amniotic injection model that allow infected mice to grow into puberty and recapitulated several symptoms of clinical ‘congenital Zika syndrome’, including decreased brain volume and thinner cortex, motor and visual dysfunction, anatomical defects in the visual circuits and cerebellum, and intracranial calcification. Our study has prognostic implication of human congenital ZIKV infection and is useful for *in vivo* evaluation of anti-ZIKV therapies.

The following are the supplementary data related to this article.

Video S1 A severely paralyzed ZIKV-infected mouse.
One ZIKV-infected mouse whose hind limb appeared to be suffering from arthrogryposis. Video S1

Video S2 Free-moving behavior of ZIKV-infected mice.
The movie was consisted of two parts. Part 1 is one female adult mouse (labeled as NO.1) that went through the surgery of intra-amniotic injection of mock and her offspring at P20. The motor function of the juvenile mice appeared to be normal. Part 2 is one female adult mouse (labeled as NO.1) that went through the surgery of intra-amniotic injection of ZIKV and her offspring at P20.The walking posture of most juvenile mice (one of them labeled as NO.2) was very different from that of mock mice. Video S2

Supplementary figures Image 1

## Conflicts of Interest

The authors declare no competing financial interests.

## Author Contributions

L.L. and J.Z. conceived the research and designed the experiments. L.L., J.Z., and S.J. supervised the project. L.C., P.Z. and E.C. performed the experiments and analyzed the data. L.C., P.Z., E.C., H.Y., L.L. and J.Z. wrote the first draft of the manuscript and prepared the figures. J.Z., L.L., S.J. and other authors discussed and contributed to the writing of the manuscript.

## Acknowledgments & Funding Sources

J.Z. thanks the following funding agencies for supporting this work: the National Natural Science Foundation of China (31271158, 31421091 and 31422025), the Young 1000 Plan and Ministry of Science and Technology of the People's Republic of China (2015AA020512). L. L. and S. J. thank the following funding agencies for supporting this work: Ministry of Science and Technology of the People's Republic of China (2015AA020930, 2016YFC1202901 and 2016YFC1201000) and the Shanghai Rising-Star Program (16QA1400300). We thank Dr. Boyin Qin for aid in micro-CT data collection and Dr. Qian Huang for aid in behavioral data analysis.
