# Supplementary figures and images for: Visual and Motor Deficits in Grown-up Mice with Congenital Zika Virus Infection
Source: eBioMedicine. 2017 Apr 24;20:193–201. doi: 10.1016/j.ebiom.2017.04.029 (PMC5478201; doi:10.1016/j.ebiom.2017.04.029)

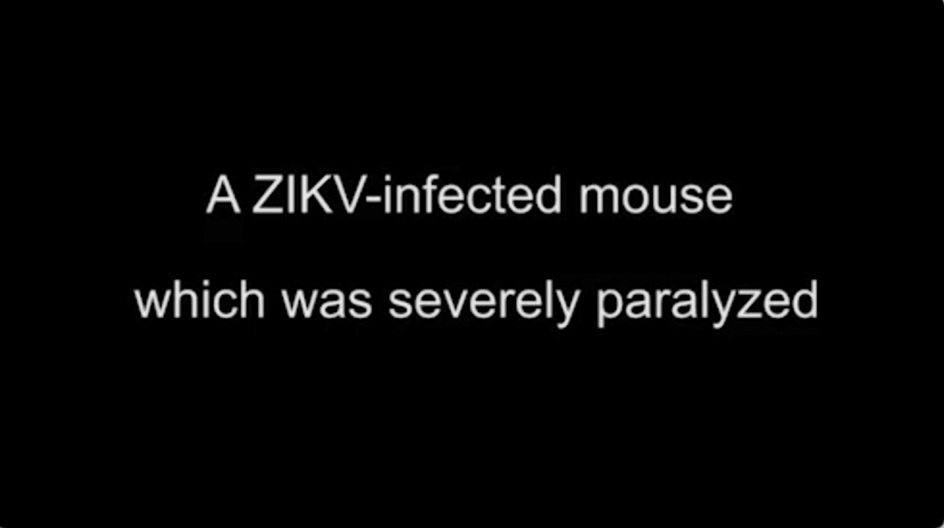

Supplement: Video S1 — A severely paralyzed ZIKV-infected mouse. One ZIKV-infected mouse whose hind limb appeared to be suffering from arthrogryposis. [file mmc1.jpg]

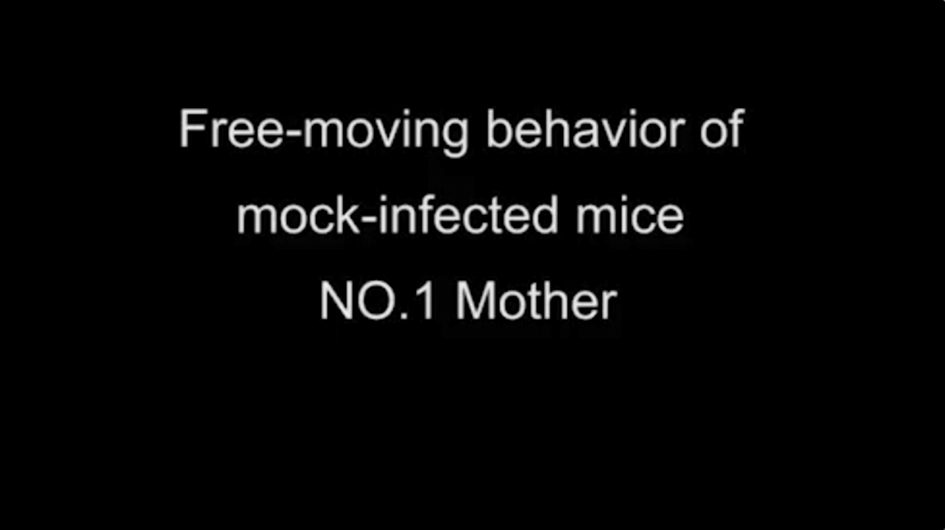

Supplement: Video S2 — Free-moving behavior of ZIKV-infected mice. The movie was consisted of two parts. Part 1 is one female adult mouse (labeled as NO.1) that went through the surgery of intra-amniotic injection of mock and her offspring at P20. The motor function of the juvenile mice appeared to be normal. Part 2 is one female adult mouse (labeled as NO.1) that went through the surgery of intra-amniotic injection of ZIKV and her offspring at P20.The walking posture of most juvenile mice (one of them labeled as NO.2) was very different from that of mock mice. [file mmc2.jpg]
