# Supplementary material for: Visual and Motor Deficits in Grown-up Mice with Congenital Zika Virus Infection
Source: eBioMedicine. 2017 Apr 24;20:193–201. doi: 10.1016/j.ebiom.2017.04.029 (PMC5478201; doi:10.1016/j.ebiom.2017.04.029)
Supplement: Supplementary file 3 — Supplementary figures [file mmc3.docx]

**Supplemental Figures and Legends**


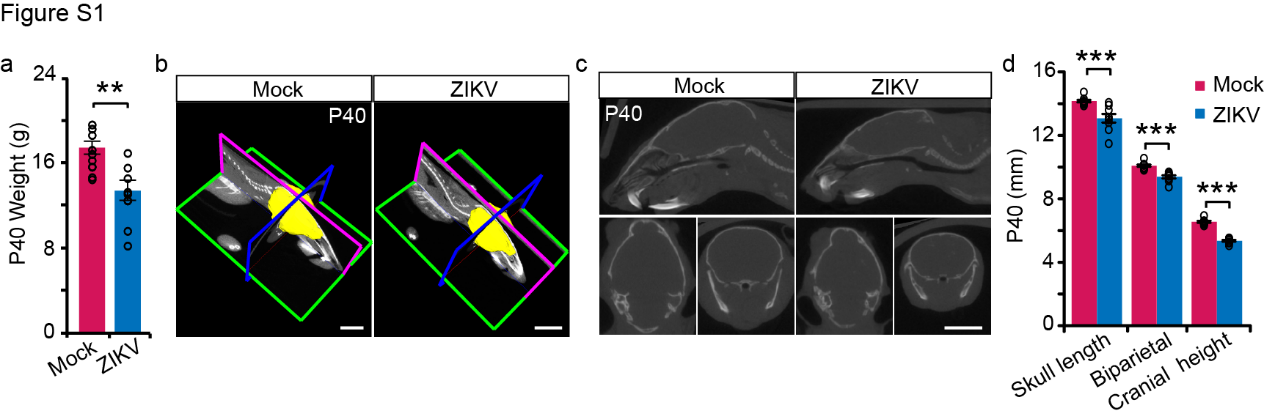


**Figure S1. Zika infection in C57 mice at P40.**

(**a**) Body weight of mock- and ZIKV-infected mice. (**b**) Micro-CT images showing brains of mock- and ZIKV-infected mice. Yellow color highlighted the reconstructed 3D brain from Micro-CT images. Scale bars, 5 mm. (**c**) Largest sagittal, coronal and horizontal Micro-CT images of mock- and ZIKV-infected mice. Scale bar, 5 mm. (**d**) Skull length, biparietal and cranial height measured from (c). All the data showed mean ± SEM. n_mock_ = 10 mice, n_ZIKV_ = 9 mice, ****P* < 0.001. Black circles represented raw data.


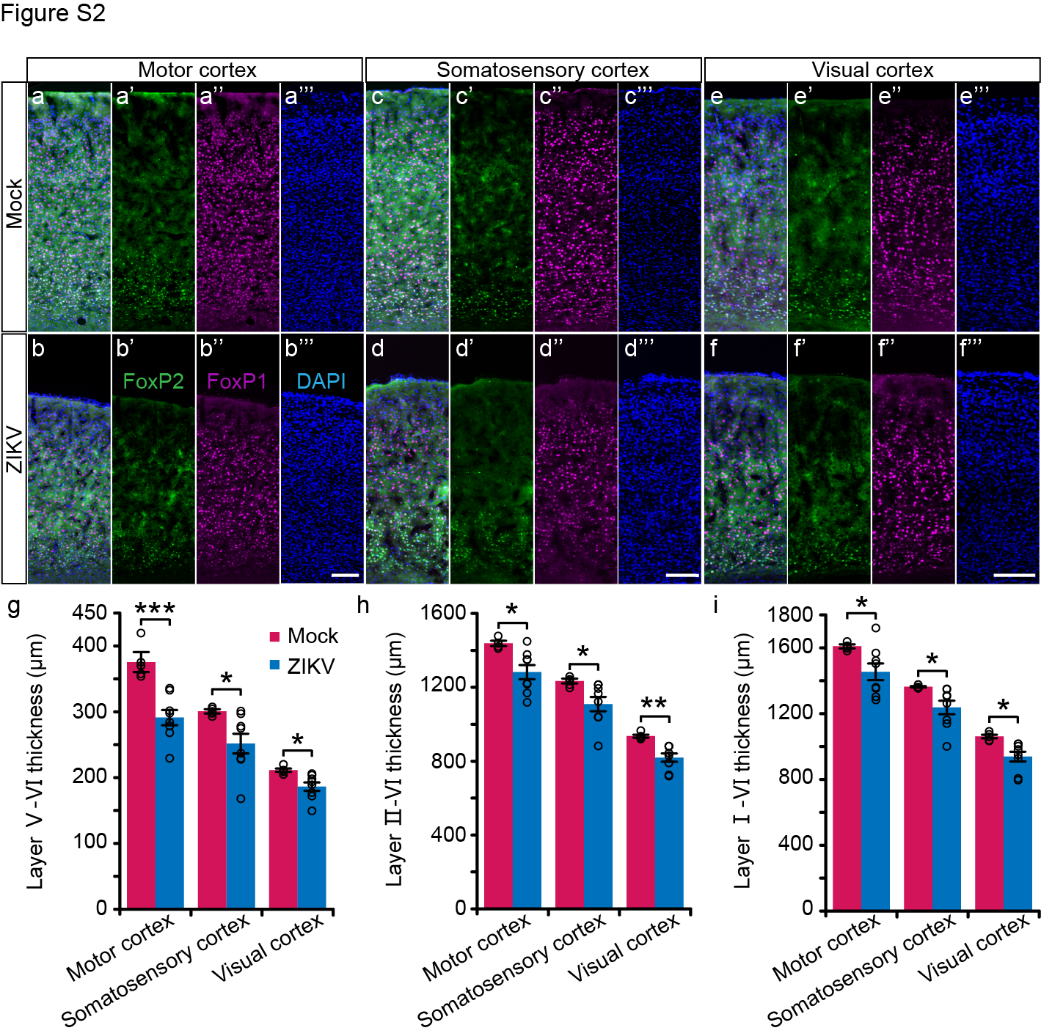


**Figure S2. Cortical defects in ZIKV-infected mice.**

FoxP2 (green), FoxP1 (magenta) and DAPI (blue) staining in the motor cortex (**a**-**b’’’**), somatosensory cortex (**c**-**d’’’**) and visual cortex (**e**-**f’’’**) of mock- and ZIKV-infected mice. Scale bars, 200 μm. (**g**-**i)** Thicknesses of layer V-VI (g), II-VI (h) and I-VI (i) in motor cortex, somatosensory cortex and visual cortex. All the data showed mean ± SEM. n_mock_ = 6 mice. n_ZIKV_ = 9 mice. ****P* < 0.001, ***P* < 0.01, **P* < 0.05. Black circles represented raw data.

**
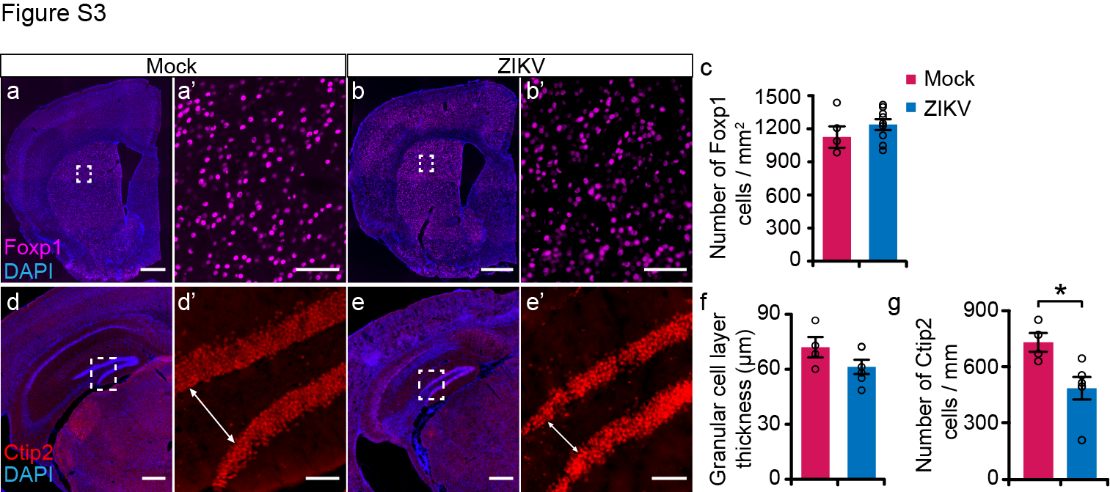
**

**Figure S3. ZIKV-infection does not cause structural changes in striatum but in hippocampus.**

(**a** and **b**) Foxp1- and DAPI-stained striatum of mock- and ZIKV-infected mice. **(c)** The number of Foxp1 cells in 1 mm^2^ area of a 30 μm thick brain slice. n_mock_ = 5 mice. n_ZIKV_ = 9 mice (**d** and **e**) Ctip2 and DAPI-stained hippocampus of mock- and ZIKV-infected mice. Scale bars, 500 μm. (**a’**, **b’**, **d’**, **e’**) High magnification images of the inset areas in (a, b, d and e). Scale bars, 100 μm. Insets in (d’, e’), white arrows indicate the width of dentate gyrus of hippocampus. **(f)** The thickness of granular cell layer expressed Ctip2 in dentate gyrus. n_mock_ = 4 mice. n_ZIKV_ = 6 mice **(g)** The number of Ctip2 cells per 1 mm length from middle position in dentate gyrus. n_mock_ = 4 mice. n_ZIKV_ = 6 mice. **P* < 0.05. Black circles represented raw data.


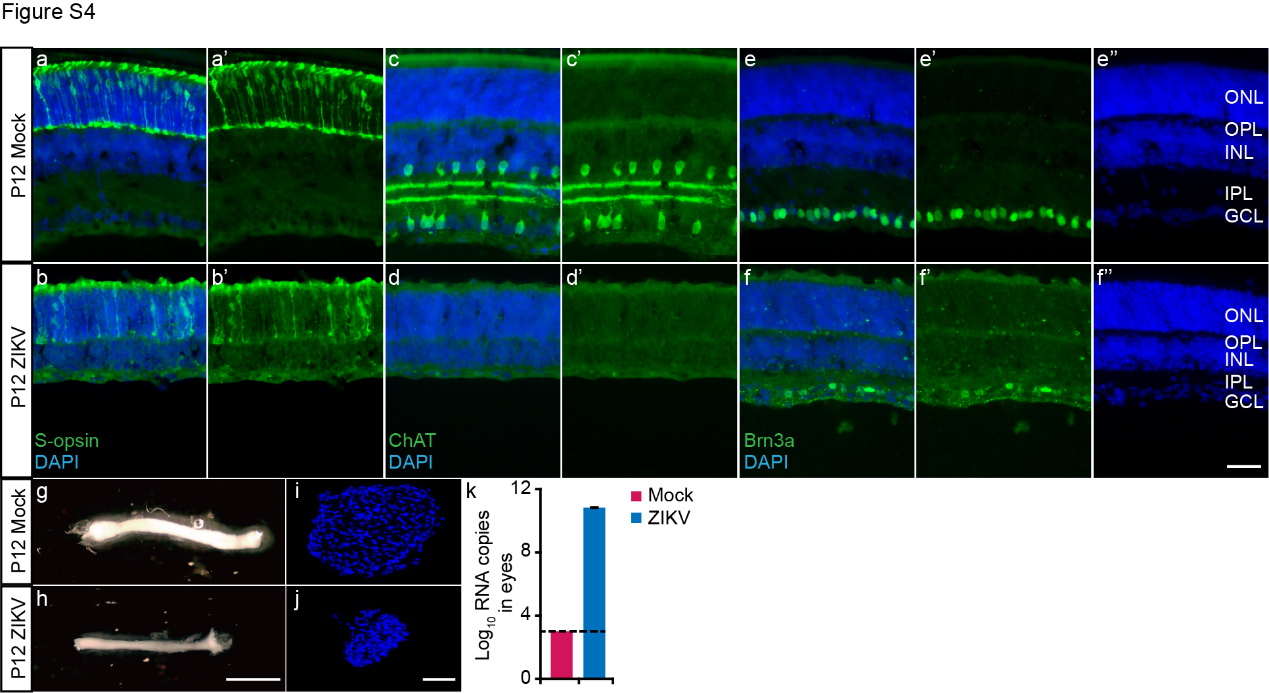


**Figure S4. Defects in the retina and optic nerve of ZIKV-infected mice at P12.**

(**a**-**b’**) S-opsin (green) and DAPI (blue) stained retinas of mock- and ZIKV-infected mice. (**c-d’**) ChAT (green) and DAPI (blue) stained retinas of mock- and ZIKV-infected mice. (**e-f”**) Brn3a (green) and DAPI (blue) stained retinas of mock- and ZIKV-infected mice. Scale bar, 50 μm. (**g** and **h**) white light images of whole mount optic nerves of mock- and ZIKV-infected mice. Scale bar, 1 mm. (**i** and **j**) DAPI (blue) stained cross-sections from optic nerves of mock- and ZIKV-infected mice. Scale bar, 100 μm. (**k**) Viral RNA copies determined by real-time PCR of whole eyes (P8) in mock- and ZIKV-infected mice. Dotted lines represent limits of detection (n_mock_ = 4 mice, n_ZIKV_ = 4 mice).
